# MicroRNAs and Autophagy: Fine Players in the Control of Chondrocyte Homeostatic Activities in Osteoarthritis

**DOI:** 10.1155/2017/3720128

**Published:** 2017-06-21

**Authors:** Stefania D'Adamo, Silvia Cetrullo, Manuela Minguzzi, Ylenia Silvestri, Rosa Maria Borzì, Flavio Flamigni

**Affiliations:** ^1^Dipartimento di Scienze Biomediche e Neuromotorie, Università di Bologna, Bologna, Italy; ^2^Dipartimento di Scienze Mediche e Chirurgiche, Università di Bologna, Bologna, Italy; ^3^Laboratorio di Immunoreumatologia e Rigenerazione Tissutale, Istituto Ortopedico Rizzoli, Bologna, Italy

## Abstract

Osteoarthritis (OA) is a debilitating degenerative disease of the articular cartilage with a multifactorial etiology. Aging, the main risk factor for OA development, is associated with a systemic oxidative and inflammatory phenotype. Autophagy is a central housekeeping system that plays an antiaging role by supporting the clearance of senescence-associated alterations of macromolecules and organelles. Autophagy deficiency has been related to OA pathogenesis because of the accumulation of cellular defects in chondrocytes. Microribonucleic acids (microRNAs or miRs) are a well-established class of posttranscriptional modulators belonging to the family of noncoding RNAs that have been identified as key players in the regulation of cellular processes, such as autophagy, by targeting their own cognate mRNAs. Here, we present a state-of-the-art literature review on the role of miRs and autophagy in the scenario of OA pathogenesis. In addition, a comprehensive survey has been performed on the functional connections of the miR network and the autophagy pathway in OA by using “microRNA,” “autophagy,” and “osteoarthritis” as key words. Discussion of available evidence sheds light on some aspects that need further investigation in order to reach a more comprehensive view of the potential of this topic in OA.

## 1. Introduction

Osteoarthritis (OA), a chronic degenerative disease of the articular cartilage, is the most common form of arthritis, affecting millions of people worldwide, with a prevalence as high as 60% in men and 70% in women above 65 [1]. OA mainly affects the major joints such as the knee and hip [2] and heavily impacts on life quality [3]. Therefore, OA represents a major burden for the National Health Systems and is expected to rise in Western countries with aging of the population.

Age is indeed one of the major risk factors for OA, but the hypothesis of a mechanical pathogenesis as dependent on “wear and tear” or “overload” is questioned by the evidence that OA may also affect non-weight-bearing joints, such as the hands. The risk of hands OA is more than doubled in obese patients, in keeping with the relevance of a systemic inflammation status [4] that compromises joint tissues. It is indeed recognized that OA is a disease with multifactorial etiology including biochemical or systemic factors (genetics, aging, dietary intake, oestrogen use, bone density, and metabolic syndrome) and biomechanical causes (muscle weakness, obesity, joint laxity, and injury) [5, 6].

The final common effect is the loss of cartilage integrity, due to the defective homeostatic balance between extracellular matrix (ECM) synthesis and degradation by the chondrocytes, the unique cells inside cartilage. Chondrocytes are indeed responsible for cartilage homeostasis through a very tight regulation of ECM turnover and recycling of damaged components.

Healthy articular cartilage homeostasis guarantees the so-called “maturational arrest” of chondrocytes and prevents their progression towards hypertrophy and terminal differentiation [7]. Instead, OA is characterized by a loss of this chondrocyte maturation block, that is, essentially “age”-related in human and other species, according to life expectancy [8], although comorbidity factors may anticipate the “age” of maturation block failure.

Aging and obesity are associated to a systemic oxidative and inflammatory status [4, 9] that can impact on chondrocyte health via mitochondria targeting. The oxidative stress condition can result from an imbalance between the production of reactive oxigen species (ROS) in mitochondria and cell ROS scavenging systems, comprising superoxide dismutase (SOD), catalase, glutathione peroxidase, glutathione reductase, and reduced glutathione.

Mitochondrial pathology has been recently recognized as having a pivotal role in OA [10] and ROS produced by dysfunctional mitochondria are able to boost cellular signalling and matrix catabolism [10]. Moreover, the turnover of damaged mitochondria via autophagy is impaired in aged and OA cartilage. Autophagy can be defined as a quality control system able to preserve the efficiency of cell activities through the removal of damaged or aged cell components such as organelles and proteins. The possibility of discarding/recycling damaged organelles is pivotal in tissue maintenance, particularly in postmitotic conditions. In particular, mitochondria may be the target of oxidative stress, and on the other hand, when aged or injured, they become defective in energy production and generate ROS at a higher rate. Autophagy failure contributes to OA pathogenesis and is responsible for the accumulation of cellular defects in chondrocytes [11, 12]. Indeed, even before the occurrence of structural damages, aged cartilage features a decrease in critical autophagy genes and an increase in mTOR, an autophagy repressor. The correlation between cartilage health and autophagy has also been confirmed with functional genomic studies [13, 14].

Cytokines, growth factors, and ECM component by-products trigger intracellular signals able to regulate chondrocyte metabolic activity and to switch on a proinflammatory and catabolic scenario. Indeed, the presence of many inflammatory mediators (such as interleukin 1 (IL-1), IL-6, IL-7, IL-8, and tumour necrosis factor-*α* (TNF-*α*)) points at OA as a low-grade inflammatory disease much more than what was initially thought [4, 15]. Inflammatory cytokines lead to cartilage destruction through activation of nuclear factor *κ*B (NF-*κ*B) [16], phosphatidylinositol 3-kinase (PI3K)/AKT, and transcription pathways [17] and induce the upregulation of major catabolic enzymes. The complex cartilage ECM is at first cleaved by aggrecanases, belonging to the “a disintegrin and metalloproteinase with thrombospondin motifs” ADAMTS family. Then, the collagen becomes accessible to the matrix metalloproteinases (MMPs). The OA cartilage shows a high expression of MMP-13, the major type II collagen (COL-2) degrading MMP, which instead is absent in healthy tissue. The pivotal pathogenetic role of MMP-13 activity has also been pointed out by functional genomics studies [18]. In the above described scenario, the outcome of articular cartilage degeneration depends on the balance between inflammatory signalling pathways and other homeostatic molecular systems, such as AMP-activated protein kinase (AMPK) and sirtuin-1 (SIRT-1) that counteract oxidative stress and inflammation [19] and also exert a pivotal role in metabolic stress and autophagy management [20]. SIRT-6 has also recently emerged as key factor in cartilage homeostasis, being decreased in both OA and aged cartilage [21].

Whatever the initial trigger, OA progression is sustained by profound changes of the epigenetic control of gene expression and transcription factors. This leads to marked changes of target gene expression in joint tissues in association with an altered methylation status of the genome [22]. In this regard, microribonucleic acids (microRNAs or miRs) are an abundant, evolutionary conserved subfamily of short noncoding RNAs (22–25 nt) acting as potent posttranscriptional regulators through target recognition modules. To date, 1881 sequences of precursor miRs and 2588 mature miRs have been identified in human cells and uploaded in the main web databases miRBase based on the new human genome assembly (GRCh38) released. Computational predictions, tools also available in many others web databases (TargetScan, miRWalk2.0, and miRanda), unveil that more than 50% of all human proteins are under potential regulation by miRs. Indeed, an altered epigenetic signature of microRNAs drives both OA onset and progression. Most of these miRNAs are directly regulated by major OA risk factors, including aging, mechanical stress, and inflammation and are able to affect homeostatic mechanisms [23].

Despite the shared final pathogenic mechanisms of the disease, OA patients present a high variability in etiologies, comorbidity factors, clinical assessment, and involvement of the other joint tissues. This hampers the definition of a useful patient stratification for both research purposes [24] or for personalized therapy. Indeed, there is an urgent need of disclosing new diagnostic and prognostic biomarkers as well as targets for really disease-modifying therapies. A complete understanding of the molecular mechanisms that keep articular cartilage homeostasis becomes critical in advancing in this direction.

## 2. MicroRNAs and OA

### 2.1. Biogenesis and Mode of Action

As initially found for *C. elegans* lin-4 and let-7, most of the currently annotated miR genes lie in introns of protein coding or noncoding genes; these miRs can occur alone or in a cluster of several miRs and are thought to be regulated by the same promoter of host genes and likely generated from the host intron. As expected, the expression level of the “host” gene mRNA is positively correlated with that of the miR under study because they share the same transcriptional regulation and function. On the other hand, the expression level of the “target” gene mRNA is negatively correlated [25]. Based on a “coherence of function,” it is becoming clear that miRs represent a tool to support the expression of host genes, while repressing the expression of antagonistic genes. However, it has been also found that a “host” gene mRNA can harbour both an intronic miR and a predicted seed sequence of this miR in its 3′ UTR [26].

The long RNA precursor with a single or several stem loops is called primary (pri)-miR. Then, pri-miR undergoes a cleavage by a miR processor composed of DROSHA (a highly conserved RNase type III) and DGCR-8 (DiGeorge syndrome critical region 8) in the nucleus. This complex generates a shorter hairpin structure, called pre-miR, of 70–100 nt that is transferred through EXPORTIN-5 to the cytoplasm, where it undresses of the loop by another RNase III, DICER, to form the double-stranded (ds) miR duplex. This ds-RNA is 22 nt in length and is composed of the mature miR and the so-called passenger miR. Generally, but not always, the latter (3′end, called passenger strand and usually shown with an asterisk) is degraded and the mature miR (5′end-thermodinamically less stable) forms the RNA-induced silencing complex (RISC) alongside with the main components, Argonaute proteins (AGOs). In this way, a specific miR addresses RISC towards specific mRNA cognate targets by matching them. Hence, miRs negatively modulate the bioavailability of mRNA targets [27]. As taps are able to regulate a flux intensity, miRs act by finely tuning protein output.

The base-pairing sequence, called seed sequence, is 2–8 nt long and lies at the 5′end of the mature miR. As mentioned above, the mechanism of action is mainly exerted by the matching of the miR to the mRNA 3′ UTR, but alternative bindings to the coding portion or to the 5′ UTR have been confirmed [28]. The repressed target mRNAs and miRs aggregate in cytoplasmic foci, called processing bodies (P-bodies) where mRNA is decayed or stored with RNA decay factors, such as AGO family members, deadenylases and GW182 [27, 29, 30]. This process, well known as RNA interference, has been discovered by Jorgensen and colleagues in 1990 [31] and characterized by Fire and Mello in 1998 with a first study in *C. elegans* showing that dsRNA is much more potent at inhibiting gene expression than antisense RNA [32]. This major breakthrough sets the stage for understanding the role of miRs in development and gene regulation.

### 2.2. miR Expression and Role in OA

Although the function of miRs needs further and deeper investigations, their involvement in cartilage and chondrocyte physiology has been established. Indeed, the importance of a successful miR processing machinery has been reported in cartilage homeostasis by means of tissue-specific knockout animal models. Kobayashi et al. showed skeletal growth defects and premature death in DICER-deficient chondrocytes derived from *DICER*-null mice. Since DICER composes the miR processor, this finding unveils the fundamental role of miRs in chondrogenesis and bone development [33]. The latter has been further confirmed by similar results achieved in mice with DROSHA and DGCR-8-deficient COL-2*α*1-expressing cells [34].

Research on the role of miR in OA pathogenesis started in 2008 with the work of Iliopoulos and colleagues who investigated the differential expression of 365 miRs in articular cartilage derived from OA patients compared with that from normal patients without a history of joint disease [35]. They reported that 16 miRs were deregulated in OA versus normal cartilage. In particular, 9 miRs were upregulated and 7 downregulated. These microarray-derived results were confirmed by real-time PCR and northern blot assay. Furthermore, a very interesting discovery was that some of these miRs showed a significant correlation with patient body mass index (BMI), opening a new window on the involvement of miRs in obesity and inflammation [35].

More recent research has focused to define an “OA signature” of circulating miRs. They are supposed to derive from several sources or events, including cartilage destruction, chronic inflammation and apoptosis, or from joint cells as a mean for establishing intercellular and intracellular communication. miRs are able to circulate in plasma and other body fluids (e.g., synovial fluid) associated to proteins, such as AGOs, or embedded in microvesicles, named exosomes. Because of their stability in body fluids, they can represent useful diagnostic or prognostic biomarkers. Beyer and colleagues were the first to investigate the potential of circulating miRs as predictors for OA and among 12 miRs identified let-7e as a promising candidate to predict OA risk because of its age-, sex- and BMI-independent association with OA [36]. Another study reported the expression profiling of circulating miRs in plasma from OA knee patients with early or intermediate (radiological score 2 or 3) OA and with a BMI <27. The study disclosed a panel of 12 miRs (miR-16, -20b, -29c, -30b, -93, -126, -146a, -184, -186, -195, -345, and -885-5p) that are overexpressed in OA compared with those in healthy subjects [37]. These findings obtained in early OA patients are “closer” to disease pathogenesis and are therefore valuable in order to identify candidate biomarkers for an early diagnosis. This could allow the use of therapeutic strategies at a stage where disease-modifying drugs could be effective in blocking or reverting disease progression. On the other hand, the pattern of circulating miR could be different in patients with advanced or end-stage OA.

The most common methods used for large-scale detection of miRs include hybridization-based microarray platforms and next-generation sequencing analysis. The major advantages of these methods consist in high throughput profiling data and therefore cost effectiveness. Despite the high quality of several studies focused on miRs in OA, it is however difficult to find consistent, shared outcomes. This discrepancy may be mostly due to the variety of the study design, with particular emphasis on patient stratification and comorbidity factors and also to different specimen sources, choices of control sources, different brand of chips, and lack of parameters to compare results from different platforms and different bioinformatic approaches and statistical analyses to process the results. On the other hand, a different, complementary approach is represented by functional studies focusing on a target gene and a putative targeting miR, usually quantified by real-time quantitative polymerase chain reaction (RT-qPCR) analysis. This is required to reliably identify the targets and provide information in support of their use in diagnosis, prognosis, or therapy.

The best characterized microRNA in OA pathology is miR-140, whose gene is harboured between exons 16 and 17 of the E3 ubiquitin protein gene *WWP2* on chromosome 8 in mouse and chromosome 16 in human genome. Tuddenham et al. reported a study on miR-140 regarding its expression in cartilage in murine embryos and its direct target, histone deacetylase 4 [38]. Later, Miyaki et al. performed a gene expression profiling through microarray technology and validation phase using RT-qPCR in human articular chondrocytes (hAC) and human mesenchymal stem cells (hMSCs). They demonstrated that miR-140 expression in MSC cultures increased in parallel with the expression of *SOX9* and *COL2A1* during chondrogenesis, and this expression was significantly reduced in OA tissue compared to that in normal cartilage. Moreover, they also showed that IL-1*β* is able to inhibit miR-140 expression in chondrocytes [39]. miR-140 is also able to target *ADAMTS5* [40] and *MMP13* [41] fundamental matrix-degrading enzymes in OA development. Therefore, miR-140 is an example of a pathogenic miR for OA, crosscutting in all OA subtypes and affecting both cartilage and the subchondral bone. Indeed, its expression resulted in altered murine models of OA induced by aging, mechanical stress, and inflammation. Moreover, miR-140 defective mice showed an altered balance between catabolic and anabolic enzymes in cartilage and spontaneously developed early OA alterations leading to loss of proteoglycans and articular fibrillation [23, 39, 40]. The reduction of miR-140 levels in many microarray data set of OA models, including human OA cartilage [35, 39], confirms that this miR is associated with OA pathogenesis. Indeed, miR-140 is a target of key chondrogenic transcription factors and functions as a central hub of the differentiation program tuning the expression of chondrocyte growth factors, key signalling proteins and matrix degrading enzymes [42]. Although most initial studies focused on the miR-140 role in cartilage physiology and pathology, later studies revealed that other miRs may act as important posttranscriptional regulators of key pathways involved in OA pathogenesis. A list of the main differentially regulated miRs in relation to OA is given in Table 1. Jones et al. showed that the overexpression of miR-9, miR-98, or miR-146 in isolated human chondrocytes suppressed IL-1*β*-induced TNF-*α* production and that miR-9 was able to modulate MMP-13 secretion [43]. This was the first study to report altered miR-9 levels in OA and prompted more researchers to investigate the miR-9 role and its putative targets [44-46]. Makki et al. demonstrated that miR-9 promotes IL-6 induction by decreasing M-phase inducer phosphatase1 (*MCPIP1*) expression [46]. These authors described the same mode of action also for miR-139 overexpressed in OA [47]. Then, Akhtar et al. reported that IL-1*β* suppressed miR-27b expression that, in turn, resulted inversely correlated to MMP-13 production, so as to identify the linear IL-1*β*/miR-27b/MMP-13 axis [48].

miRs are also deregulated in aging, affecting cellular mechanisms such as senescence, mitochondrial homeostasis, oxidative defence, and DNA repair. MicroRNA expression observed during age have a specific deregulated pattern depending on cell and tissue type, despite the existence of some common age-related miRs that control general cellular functions [49]. OA cartilage shows reduced expression of miR-24 that targets *P16INK4*, a factor able to affect cell cycle and senescence. Selective inhibition of P16INK4 delays age-related diseases [50], whereas its upregulation in OA is responsible for increased MMP-1 expression, thus disclosing the dual ability of miR-24 to control both cell senescence and ECM remodelling [51].

Age-related miRs target factors affecting cell capability to counteract stress (e.g., oxidative stress) and senescence-associated factors. miR-34a is able to regulate at the same time the mitochondrial function and oxidative stress, DNA damage and P53 pathway, and apoptosis and differentiation. miR-34a is upregulated in samples derived from OA patients [52] and increased in chondrocytes after IL-1*β* exposure in a rat model. Accordingly, miR-34a silencing reduces apoptosis by reverting the IL-1*β*-upregulation of inducible nitric oxide synthase [53]. miR-34a is implicated in stress-dependent senescence, being a direct target of *P53* and in its turn targeting *SIRT1* [23, 49]. The role of this loop has recently been elucidated in OA development [52]. It has been demonstrated that miR-34a induces apoptosis and reduces proliferation of human chondrocytes by directly targeting *SIRT1*. Indeed, SIRT-1 reduction observed in OA patients and in vitro experiments is responsible for reduced P53 deacetylation and consequently reduced BCL-2 and increased BAX levels. Noteworthy, the therapeutic potential of “miR-34a targeting” has been confirmed in a rat model of OA where intra-articular injection of lentiviral vector encoding anti-miR-34a led to a significant delay in disease progression [52]. Sirtuins are enzymes in charge of regulating resistance to stress. miR-34a at the same time targets *SIRT1* and *SIRT6*; the latter recently emerged as a key regulator in chondrocyte homeostasis [54]. The negative correlation between SIRT-6 and miR-34a expression uncovered in squamous cell differentiation could be translated to aging and other tissues [55]. Indeed, *SIRT6* knockout mouse shows a dramatic aging phenotype and spontaneously develops a progeroid syndrome. The degenerative phenotype of these mice also affects the bones and it is characterized by alterations of transcription, genomic instability, and above all by impairment of DNA repair [56]. In addition, SIRT-6 prevents senescence and DNA damage in human chondrocytes [57]. Interestingly, miR-34a impacts on mitochondrial stress being able to target the antioxidative enzyme *TXNRD2*. The only other age-related miR involved in mitochondrial control is miR-335 which targets *SOD2*. Both these enzymes are crucial in the control of ROS neutralization [58].

Most of recent studies have shown the ability of one or two miRs to simultaneously target some factors or pathways that are dysregulated in OA pathogenesis. For instance, miR-127-5p was shown to target osteopontin (*OPN*), an important regulator of OA-related factors, such as MMP-13, TIMPs, and ADAMTS-4, in human chondrocytes [59]. miR-26a-5p was found to be suppressed by IL-1*β*-mediated NF-*κ*B activation, thereby allowing induction of its direct target *iNOS* in cartilage [60]; in addition, miR-26a, alongside with miR-26b, was found to modulate NF-*κ*B p65 translocation via repression of karyopherin subunit alpha 3 (*KPNA3*) [61]. Therefore, downregulation of some miRs may contribute to the pathogenesis of OA via promotion of the NF-*κ*B signalling pathway. Interestingly, miR-33a, one of the master regulators of cholesterol and fatty acid metabolism, was also shown to modulate cholesterol homeostasis in chondrocytes through the TGF*β*1/AKT/SREBP-2 pathway, as well as cholesterol efflux-related genes *ABCA1* and *APOA1* [62].

Although in the last decade numerous studies have been carried out with the purpose of detailing new miR-targets related to OA changes, few recent findings provide novel insights on a new role for miR in system biology, with particular reference to the OA field. Outside the box, Li et al. reported a synergistic collaboration of two miRs, miR-140 and miR-29, able to reverse the increase in IL-1*β*-stimulated MMP-13 and TIMP-1 levels and, in turn, to rescue type II collagen in an in vitro model of OA [63]. The preliminary potential can be set on the basis of the useful miR synergy score comprising two independent parameters, the target similarity score (TSS) and the protein interaction score (PIS) [64]. Recently Kang and colleagues investigated the role of miR-23a-3p in OA progression by directly targeting small mother against decapentaplegic 3 (*SMAD3*). More interesting, they identified a hypomethylated status of CpG islands in the promoter region of miR-23a-3p in OA patients, thus accounting for the higher levels of this miR in OA cartilage compared with those in normal tissues [65].

Although these studies are far behind the possibility of translating their findings into clinical applications, nevertheless, they represent the basis to develop future therapeutical strategies aimed to affect dysregulated pathways by employing stable synthetic miRs or anti-miRs.

## 3. Autophagy in OA

### 3.1. Phases and Actors of Autophagy Process

Based on the type of cargo delivery, three different forms of autophagy have been described in mammals: macroautophagy, microautophagy, and chaperone-mediated autophagy, all of them characterized by proteolytic degradation of cytosolic components at the lysosome. “Macroautophagy” is a bulk degradation of cytosolic portions after fusion with a lysosome. This is the most prevalent and studied form of autophagy, and hereafter, we refer to macroautophagy simply as autophagy. The first step of this process is represented by the isolation of a membrane, named “phagophore”, from the endoplasmic reticulum (ER) and/or the trans-Golgi and endosomes, which engulfs the cargo by stretching the ends around and thereby incorporating it in a double-membrane “autophagosome”. Then, the stuffed autophagosome is ready to fuse with the lysosome thus forming the “autolysosome” whose cargo is finally digested by the lysosomal acidic hydrolases [66, 67]. The autophagy machinery involves several autophagy-related proteins (ATGs) participating in each stage of this dynamic process [68].

Although autophagy is active in the cell under basal conditions to guarantee the replacement of old with fresh, better quality components, several key proteins influence this process responding to many stimuli [20]. One of the main sensors of nutrient and energy status is the mammalian target of rapamycin (mTOR) kinase. It can be activated by the AKT-PI3K pathway when nutrients and growth factors, such as insulin, are available so as to stimulate cell growth by increasing protein translation [69]. mTOR forms two different complexes, alternatively interacting with either RAPTOR to form TORC-1, directly involved in autophagy inhibition, or with RICTOR to form TORC-2, mainly influencing cytoskeleton organization and cell survival [70, 71]. Once activated, TORC-1 suppresses the most upstream autophagy protein complex that comprises unc-51-like kinase-1 (ULK-1), ULK-2 (corresponding to ATG1 in *C. elegans*), ATG-13 and focal adhesion kinase (FAK) family-interacting protein of 200 kDa (FIP-200) [72]. This first complex is required for phagophore formation because it triggers the activation of the BECLIN-1 complex including vesicular protein sorting-34 (VPS-34), P150, ATG-14L, UVRAG, and RUBICON [73].

Two ubiquitin-like conjugation systems are responsible for autophagosome elongation and maturation steps: the ATG-12–ATG-5–ATG-16L1 complex and the microtubule-associated protein light chain-3- (LC-3-) phosphatidylethanolamine (PE) machinery. In the first case, ATG-7 activates ATG-12 which, through an E2-like ubiquitin carrier protein, covalently binds ATG-5. This complex finally associates with ATG16L. LC-3, the mammalian homologue of ATG-8 in yeast, undergoes an ATG-4-mediated proteolytic cleavage resulting in the formation of LC-3I, a cytosolic form able to bind ATG-7, subsequently conjugated by ATG-3 (E2-like ubiquitin carrier) to phosphatidylethanolamine (PE) so as to generate LC-3II. This latter processed form is engaged to the phagophore membrane by means of ATG-12-ATG-5 system and it becomes responsible for the fusion between membrane ends and for cargo selection. In fact, LC-3II acts as a phagophore receptor that recognizes P62/SQSTM-1, an adaptor protein that links targets, such as polyubiquitinated protein aggregates and mitochondria, addressing them to autophagic degradation.

Another protein sharing sequence similarity with LC-3 is GABARAP (GABA(A) receptor-associated protein) [74]. The abundance of members in this ATG-8 homologue family is due to their specific involvement in different types of autophagy (e.g., aggrephagy, mitophagy, pexophagy, ribophagy, or xenophagy) [75].

Finally, in the maturation step, autophagosomes fuse with lysosomes to form the so-called “autolysosomes” where cargo is degraded and the resulting products are released in the cytosol in order to be recycled [76].

Other important proteins involved in the control of autophagy are AMPK and SIRT-1 both with promoting actions on this process. Low ATP levels and thus high adenosine 5′-monophosphate (AMP)/ATP ratio lead to the activation of AMPK. This kinase is able to directly phosphorylate ULK-1 [77] and negatively modulate TORC-1 [78]. Like AMPK, SIRT-1 is a sensor of cell energy status since its deacetylase activity depends on the NAD^+^ level. This enzyme promotes autophagy by directly acting on several ATGs; moreover, it can control autophagy gene expression through the transcription factor forkhead box O-3 (FOXO-3), [79, 80]. A complex crosstalk between AMPK and SIRT-1 is emerging, in keeping with the evidence that these proteins are able to influence each other in several ways [20].

Autophagy is also modulated by myo-inositol-1,4,5-trisphosphate (IP-3). This second messenger and its receptor IP-3R repress autophagy as well as regulate many biological processes like cell differentiation, growth, and apoptosis [81]. This mechanism can be suppressed in a TORC-1-independent fashion through the inhibition of inositol monophosphatase (IMPase), thus reducing free inositol and IP-3 [82].

### 3.2. Evidence of Autophagy Modulation in OA

In vitro and in vivo studies showed that autophagy dysregulation is related to several disorders, including metabolic diseases [83], neurodegenerative pathologies [84] cardiovascular diseases [85-87], and cancer [88]; defective or excessive autophagy can address towards pathogenesis; however, a change in autophagic flux may simply occur without having a causal role in the disease but merely as one of its manifestations.

First, studies considered autophagy as a mechanism of cellular self-destruction leading to cell death so that in some case, it has been identified as the type II-programmed cell death. In particular, Roach et al. in 2004 reported for the first time a specific variant of apoptosis in chondrocyte, called “chondroptosis”, characterized by the presence of autophagic vacuoles and increasing amount of ER membrane [89]. The colocalization of autophagy and apoptosis markers has been recently confirmed in an animal model of OA in late degenerative lesions, expression of the combination of both types of cell death [90]. On the other hand, inducers able to sustain autophagy cascade have been classified as longevity promoters, confirming the idea that autophagy may be exploited as a therapeutic target for age-related pathologies, including OA [91]. Actually, during the initial degenerative phase at the beginning of OA pathologic process, autophagy may act as a protective response to environmental stress, but during OA progression, autophagy efficiency decreases leading to cell death. Several studies reported beneficial effects of autophagy in preventing chondrocyte death, OA-like changes in gene expression, and cartilage degeneration [92-96]. Noteworthy, while investigating the role of autophagy in human chondrocytes and OA pathophysiology, Sasaki and colleagues observed that the inhibition of autophagy caused the increased expression of OA-like gene, and conversely, the induction of autophagy prevented them. Furthermore, ROS activity was decreased by induction of autophagy [95].

Defects in autophagy, reported in aged and OA-affected cartilage, include a reduced number and size of autophagosomes and this is, at least in part, related to a reduced expression of the autophagy proteins ULK-1, BECLIN-1, and LC-3 [97] and mTOR overexpression [14]. Indeed, many recent papers endorsed the importance of the mTOR pathway in cartilage regulation. In a surgically induced OA model (medial meniscus destabilization) established in mice with cartilage-specific ablation of mTOR, Zhang et al. observed increased autophagy, decreased apoptosis, and a lower level of OA catabolic factors [14]. Moreover, the reduced expression of the mTOR endogenous inhibitor REDD-1 has been found in OA and aged cartilage [98]. Another recent study showed that *TSC1* knockout, associated with hyperactivation of TORC-1, provokes an OA-related phenotype in cartilage, possibly connected to the downregulation of two receptors involved in chondrocyte proliferation and differentiation: the fibroblast growth factor receptor 3 (FGFR-3) and parathyroid hormone (PTH)/PTH-related protein (PTHrP) receptor (PPR) [99]. Located upstream mTOR, the transcription factor peroxisome proliferator-activated receptor gamma (PPAR-*γ*) regulates cartilage homeostasis, since its deficiency was found to be associated to increased TORC-1 signalling, impaired autophagy, and OA [100]. Increasing evidence identified SESTRINS (SENS) as key factors able to influence aging processes [101]. SENS are stress-induced proteins known to be involved in cell survival, DNA stability, and metabolic homeostasis. A recent paper reports that, even in chondrocytes, these molecules support cell survival under stress conditions, by inhibiting mTOR and promoting autophagy flux [102].

Insulin is one of the best known TORC-1 activators and can depress autophagy in various cell systems. Therefore, the study of the effects of this hormone in chondrocytes is particularly relevant in the OA field, since its levels increase in association with insulin-resistance, a major determinant of the metabolic syndrome. Indeed, a new OA subtype has been identified in relation to metabolic syndrome and related pathologies, such as type 2 diabetes (T2D) and obesity. Consistent with previous evidence indicating autophagy as a common deregulated pathway in both these hyperinsulinemia-related conditions and in OA, an inhibitory effect of insulin on autophagy has been demonstrated in chondrocytes in association with the increase of molecular markers of matrix degradation and inflammation. Moreover, chondrocytes derived from T2D patients showed impaired autophagy [103], consistent with the current opinion that identifies diabetes as a risk factor of OA and also suggesting a possible direct causal role. Moreover, Ribeiro et al. have recently shown that diabetic mice are more prone to developing OA features and that the treatment with the autophagy inducer rapamycin is effective at protecting cartilage tissue [104].

A close correlation between the insulin signalling and leptin is emerging, since high levels of both are common in obese patients. This adipokine, classically involved in food intake and energy expenditure modulation, has also been found overexpressed in OA [105]. In particular, lysyl oxidase-like-3 (LOXL-3), a copper-dependent amine oxidase, seems to be involved in leptin-induced mechanism of apoptosis stimulation and autophagy inhibition in chondrocytes [106].

It is known that AMPK stimulation exerts a protective effect on chondrocytes mediated by the activation of autophagy. In keeping with this notion, a decrease of AMPK activity has been found in OA cartilage while its stimulation reduces detrimental catabolic changes induced by proinflammatory stimuli [107]. In an interesting review, Liu-Bryan provided a new perspective in which AMPK and SIRT-1 are of particular importance in joint tissue homeostasis in part thanks to their autophagy-promoting activity [108]. Therefore, the search of molecules able to activate these enzymes deserves interest in the OA field.

At present, one of the therapeutic tools available to reduce cartilage inflammation and the resulting pain is represented by glucocorticoids (GC). However, besides the known side effects, these compounds may induce detrimental effects on some tissues. A recent paper has reported the effects of dexamethasone, a synthetic GC, on chondrocyte autophagy and apoptosis. The authors found that this molecule promotes autophagy flux via ROS/AKT/FOXO-3 pathway as well as apoptosis [109]. In this context, autophagy exerted a protective effect counteracting cell death, since its impairment provoked ROS accumulation and apoptosis increase in the presence of dexamethasone. In a following study, the same authors demonstrated that the prosurvival effect of autophagy is at least partially mediated by the suppression of IP-3R signalling [110].

In the light of the limited current therapeutic options, research in OA is strongly committed to the evaluation of new molecules. Based on the evidence here reviewed, a useful screening would be targeted to identify molecules able to modulate autophagy. Among these, several natural compounds classified as “nutraceuticals” have been studied [111] and some of these exert an antioxidant action thus reducing ROS and cell death. For instance, sulforaphane, derived from cruciferous aliments, exerts prosurvival and antiapoptotic effects on chondrocytes [112]. Hydroxytyrosol is another nutraceutical with proved ability to modulate autophagy and protect the cartilage [113]. This olive-derived compound promotes autophagy by stimulating SIRT-1 and increasing the transcription of P62, required for autophagic degradation [114]. Naturally occurring polyamines, which can be synthesized in cells and also introduced with the diet, are other molecules implicated in chondrocyte survival. Indeed, several papers demonstrated their role in modulation of apoptosis, hypertrophy, and terminal differentiation of chondrocytes [94, 115-117]. Among these polycations, spermidine recently became the focus of much interest in virtue of its ability to modulate autophagy as already demonstrated in various cells and tissues [118-120]. Thus, new studies are required to evaluate the possible use of spermidine to stimulate autophagy in chondrocytes and as a therapeutic or preventive agent in OA.

## 4. miR and Autophagy in OA

As aforementioned, both miR metabolism and autophagy have been widely involved in cartilage homeostasis. Indeed, many dysregulated miRs and autophagy markers turned out to be related to OA onset and progression. In the last years, numerous papers highlighted the interaction between miRs and autophagy pathway in several age-related pathologies, such as cancer and neurodegenerative diseases [121-123]. A useful classification of these “autophagic miRs” (also known as autophagomiRs) distinguishes them on the basis of their positive or negative action on the successful accomplishment of autophagy. This final effect clearly depends on miR targets. Zhu et al. were the first to report miR-30a as a negative regulator of autophagic activity by targeting *BECLIN1* [124]. Then, more miRs have been demonstrated to directly modulate different components of the autophagic machinery, at every stage of the flux: induction, vesicle initiation, elongation, and fusion. For example, *ULK1* is a target of miR-290-295, miR-20a, miR-106b, and miR-25 [125-127], while *ULK2*, exerting a redundant function in case of ULK-1 deficiency, is a direct target of miR-855-3p in response to chemotherapeutic drugs [128]. Moreover, miR-855-3p shows seed-complementary sequence to other apoptosis and autophagy-related genes, such as *MDM4*, *BCL2*, *CASPASE2*, and *CASPASE3*, leading to the understanding that these small modulators are finely inserted in the complex regulatory network of cellular processes [128]. The phase of vesicle initiation was suppressed by miR-30a/b, miR-376b, miR-17-5p, and miR-216a [129-132] inhibiting *BECLIN1* expression; by miR-152 [133] that targets *ATG14*; by miR-101 [134] downregulating *RAB5A*; and by miR-24-3p, miR-376b, miR-101, and miR-34a [130, 134-136] that modulates *ATG4*. Elongation stage was inhibited by miR-204 that directly targets *LC3* [137, 138]. Other miRs are reported as suppressors of this phase, including miR-143, acting on *GABARAPL1* [139], miR-30a [140], miR-181a [141], and miR-224-3p [142] downregulating *ATG5* and miR-17/20/93/106 [143] targeting *P62*. Additionally, miR-207, miR-320a, and miR-95 are involved in the fusion step, the latter two respectively acting on *LAMP1* and *SUMF1* (activator of cellular sulfatases) [144, 145].

In addition to directly targeting autophagy-related gene expression, miRs modulate the mTOR pathway and other key proteins, including sirtuins and AMPK and transcription factors, such as FOXOs, implicated in the regulation of autophagy. Already described as the main negative modulator of autophagy, TORC-1 is suppressed by miR-155, miR-100, and let-7 [146-148], belonging to the restricted class of proautophagic miRs. A genome-wide RNA-mediated interference screen showed that miR-19, included in the miR-17/92 cluster, targets several autophagy modulators, such as *BIM*, AMPK, phosphatidylinositol-3,4,5-trisphosphate 3-phosphatase (*PTEN*), and protein phosphatase 2A (*PP2A*), both involved in AKT regulation [149]. *AMPK* expression is also modulated by miR-137 [150]. Many miRs, including miR-34, miR-212, and miR-141, have been identified as direct repressors of *SIRT1*, thereby modulating apoptosis and autophagy [151-153]. Instead, the miR-132/212 cluster has been found to modulate autophagy through the miR-132-*FOXO3* matching [154].

**Figure 1:**
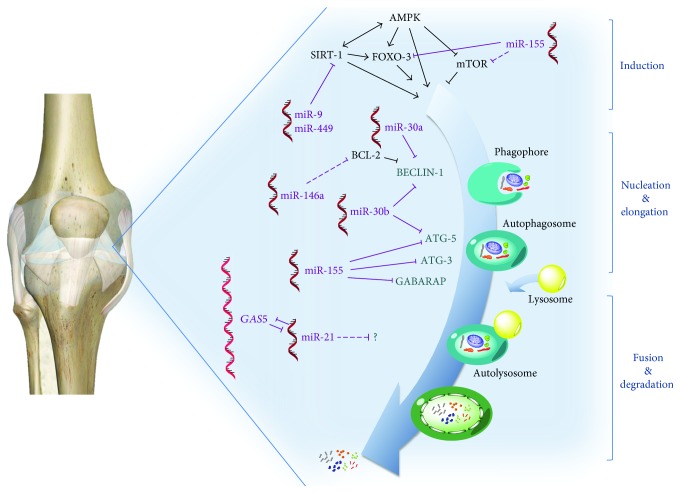
Representative drawing of the autophagy pathway modulated by key miRs in joint tissues. FOXO3 [160], SIRT1 [44, 162], mTOR [160], and BECLIN-1 [155, 157] are orchestrators of the autophagy-related gene expression or of the induction phase of autophagy that have been reported as crucial targets. Other miRs have been identified as repressors of ATG5 [155, 160], ATG3, and GABARAPL1 [160] proteins that are involved in maturation and elongation steps. Dashed lines indicate indirect effect of miRs on autophagy.

Despite a large body of evidence that demonstrates the critical role of autophagy in the OA field and the importance of several miRs as crucial modulators of some OA-related features, the molecular details of this crosstalk have not been extensively investigated in this disease. Thus, a deeper understanding of the connections between deregulated miR and autophagy impairment in OA is awaited. A summary of miR-mediated regulation of the autophagy process in OA is depicted in Figure 1.

Recently, Chen et al. have demonstrated a direct interaction between miR-30b and the 3′ UTR of both *BECLIN1* and *ATG5* mRNAs in an OA in vitro model. In particular, they identified in miR-30b a crucial modulator of the response of ATDC5 chondrocytes to TNF-*α* treatment by orchestrating the balance between the autophagic and the apoptotic processes. TNF-*α* can stimulate both pathways, but the silencing of this miR addresses cells to autophagy, whereas its overexpression promotes the cell death program. Thus, anti-miR-30b treatment can protect ATDC5 cells from apoptosis and attenuate ECM degradation through the upregulation of autophagy [155]. In keeping with the notion that a miR family generally shares an identical seed sequence and has common predicted targets [156], *BECLIN1* resulted to be also a direct target of miR-30a, a different member of the miR-30 family [157]. In the latter study, the extent of autophagy was investigated in synovial tissue specimen derived from OA and RA patients, by the assessment of BECLIN-1, LC-3, and LC-3II. An upregulation of autophagy was evidenced in RA compared to that in OA synovial tissues, where instead, apoptosis was prevailing. The authors speculate that the correlation between impaired apoptosis and enhanced autophagy in RA patients may be due to a deficiency in miR-30a expression. Zhang and colleagues conducted a study in OA primary chondrocytes focusing on the role of miR-146a in regulating autophagy under hypoxic conditions. miR-146 has been indicated as a critical regulator of autophagy by decreasing BCL-2 via HIF-1*α* under hypoxia. In particular, they demonstrated that *HIF1α* and miR-146a overexpression induced ULK-1 and ATG-5 expression in a normal 21% oxygen tension, and, conversely, *HIF1α* and miR-146 silencing reduced the expression of these autophagic proteins in cells cultured in hypoxia (0.5% oxygen tension). Thus, according to their results, miR-146a represents a chondroprotective miR, being able to promote autophagy [158]. Then, the same authors elucidated the link between miR-146a and BCL-2 and their implications on autophagy. TNF receptor-associated factor 6 (*TRAF6*) and IL1 receptor-associated kinase 1 (*IRAK1*), implicated in the NF-*κ*B-related inflammatory response, were identified as direct miR-146a targets and actual mediators of the effects exerted on BCL-2 and autophagy in OA. Hypoxia-induced miR-146a represses *BCL2* expression through TRAF-6/IRAK-1 but not SMAD-4 to promote chondrocyte autophagy [159]. Another potential regulator of autophagy is miR-155. Its seed sequence was recognized in the messenger of several autophagy-related proteins, including ATG-3, GABARAPL-1, ATG-5, ATG-2B, LAMP-2, and FOXO-3. Indeed, miR-155 was able to affect autophagy by matching some predicted cognate targets (*ATG3*, *GABARAPL1*, *ATG5*, and *FOXO3*) and other factors involved in the autophagic cascade (*ULK1*, *LC3*, and *ATG14*). Unexpectedly, miR-155 was found to inhibit mTOR activity rather than activating it, possibly by targeting *RICTOR*, a critical component of TORC-2 that induces TORC-1 activation via AKT. It can be concluded that miR-155 activity suppresses autophagy by downregulating the autophagy-related factors, independently of its effects on the mTOR pathway [160]. *SIRT1*, an important key autophagy regulator in OA, was identified as target of many miRs, including the widely studied miR-34a and also miR-449 [161, 162]. Recently, another study aimed to characterize the molecular mechanisms underlying the protective effect of hydroxytyrosol in OA found that this antioxidant promoted *SIRT1* expression and, consequently, the autophagy pathway in chondrocytes [114]. In this panorama, miR-9 was demonstrated to act as a fine modulator of its genuine target *SIRT1*, already implicated in autophagy. Moreover this miR was able to mediate OA-related changes induced by oxidative stress [44].

miR activity may be conditioned by another class of noncoding RNAs that has also been reported to modulate autophagy. Long noncoding RNAs (lncRNAs) are transcripts of about 200 nt, whose biological function and mode of action are still not completely clarified. However, in OA, they are implicated in the modulation of the autophagy pathway through the buffering of miR availability. Indeed, acting like sponges, lncRNAs can bind a miR or a set of miRs thus preventing their interaction with the mRNA targets and therefore attenuating their final effects. Song et al. first demonstrated the role of growth arrest-specific 5 (*GAS5*), a lncRNA upregulated in OA cartilage, in apoptosis and autophagy by sequestering miR-21. The authors speculated a possible mutual regulation between miR-21 and *GAS5* [163].

Noteworthy, not only the miR network can influence the autophagy pathway but also the contrary can occur. Some evidence indicates that *ATG5* silencing can induce DICER and AGO-2 accumulation, while rapamycin-increased autophagy can reduce their levels. Furthermore, DICER and AGO-2 colocalized with the selective autophagy receptor NDP-52 (also known as calcium binding and coiled-coil domain-2 (CALCOCO-2)) into autophagosomes. Interestingly, precursor and mature miRs were not found to colocalize with DICER and AGO-2, thus suggesting that only the DICER/AGO-2 complexes unloaded of miRs are targeted to autophagy degradation, leaving instead the miR-attached DICER/AGO-2 complexes free to exert their physiological function [164]. Autophagy may be required for the homeostatic balance of the miR network. This point of view, totally unexplored in OA, could potentially disclose new interesting aspects that need to be elucidated.

## 5. Conclusions and Perspectives

Emerging evidence supports the functional links that connect the autophagy pathway with the miR network in both directions, but the scientific community has paid more attention to the posttranslational modifications and protein interactions of the autophagic machinery components. Therefore, this review has focused on the latest studies with the aim of deepening our knowledge of the complicated molecular scenario where miRs, autophagy, and their connections contribute to OA pathogenesis.

Autophagy is a dynamic process that is best evaluated on the basis of the effectiveness of autophagic flux, that is, of the clearance of oxidative damage markers. A statistical assessment of autophagy as an instant shot can indeed be misleading, since senescent cells feature an overlap of autophagy, apoptosis, and senescence markers. On the other hand, literature evidence from both in vitro and in vivo studies indicated that an increase of the autophagic flux leads to the effective clearance of aged molecules and oxidative damage markers and therefore to the rescue of an anabolic phenotype in cartilage or chondrocytes. Therefore, the promotion of the autophagic pathway can represent an adaptative response of chondrocytes to several stressors, and in this way, it can protect chondrocytes from the degenerative processes occurring in OA. Hence, the modulation of autophagy by genetic manipulation, pharmacological treatment, or nutraceuticals can be a promising therapeutic strategy for OA since it has the potential to counteract both the effects of the inflammatory stimuli and the age-related defects. Indeed, the extension of known application methods of some miRs such as miR-34, miR-155, and miR-21 in therapy in other models may constitute a bottom support for their use in OA treatment and autophagy control [165].

However, the loss of autophagy efficiency often occurring in age-related pathologies needs to be investigated in the perspective of understanding its role and before beginning a treatment. Indeed, this process and its manipulation may have different outcomes depending on tissue characteristics, pathological context, and the extent of process alteration.

Based on the findings of our literature survey, the function of miR metabolism and its role in OA is at gunpoint but not yet fully understood. However, increasing therapeutic breakthrough in other diseases discloses the potential of this class of molecular targets in clinical practice also in OA. Despite the promising discoveries, this review sheds light on some aspects that need to be deeply investigated in the OA field and that allow us to hypothesize that miR tuning of cartilage biology represents an effective tool for the future management of this disease. Moreover, miR level profiling of blood or synovial fluid samples may provide important tools for novel noninvasive tests allowing for an accurate diagnosis, staging, and prediction of patients' potential response to therapy, thus constituting the basis of the long awaited “precision medicine” with the potential of reducing healthcare costs. To date, few clinical trials tested the possibility to use miRs as novel molecular biomarkers for OA diagnosis and prognosis. Beyond this application, miRs can represent potent strategies in the therapeutic field, thanks to the possibility to modulate their levels through RNA-based drugs including antisense miR oligonucleotides, miR mimics, miR sponges, and vectors expressing miR genes. These tools allow to restore or inhibit the levels of specific miRs, thus affecting entire pathways that are fundamental for tissue homeostasis, such as autophagy. Despite the great potential of this therapeutic strategy, many variables need to be carefully considered and addressed, such as delivery issues, carrier-induced cytotoxicity, and hybridization-dependent and -independent off-target effects. Nevertheless, increasing research and technological advances hold promise in other fields, such as cancer. Obviously, these studies cannot be easily translated in a different pathologic context, but they are spurring the rheumatology community to follow this approach. Indeed, this kind of gene therapy for OA may represent a true “disease-modifying” approach since it may block cartilage degeneration by targeting the key modulators of autophagy. However, further investigations of miR-based molecular therapies are necessary to shorten the distances between preclinical studies and clinical applications.

## Figures and Tables

**Table 1:** Summary of differentially expressed miRs in relation to OA discussed in the text.

MicroRNA	Targets	Pathway	Model	Reference
miR-9	MMP13	Matrix degradation	Human osteoarthritic chondrocytes	[43]
	SIRT1	Autophagy		[44]
	MCPIP1	Inflammation		[46]
miR-21	lnc GAS5	Apoptosis	Human osteoarthritic chondrocytes	[163]
		Autophagy		
miR-23a-3p	SMAD3	TGF-*β* signal pathway	Human osteoarthritic chondrocytes	[65]
miR-24	p16INK4	Cell senescence Matrix degradation	Human osteoarthritic chondrocytes	[51]
miR-26a	KPNA3	p65 translocation	Human osteoarthritic chondrocytes	[61]
miR-26a-5p	iNOS	NF*κ*B pathway	Human osteoarthritic chondrocytes	[60]
miR-26b	KPNA3	p65 translocation	Human osteoarthritic chondrocytes	[61]
miR-27b	MMP13	Matrix degradation	Human osteoarthritic chondrocytes	[48]
miR-29a	TIMP1	Matrix degradation	Mouse chondrocytes	[63]
	MMP13	Matrix degradation	Mouse chondrocytes	
miR-30b	BECN1	Apoptosis Autophagy	ADTC5 cells	[155]
	ATG5	Matrix degradation		
miR-33a	SMAD7	Cholesterol homeostasis	Human osteoarthritic chondrocytes	[62]
	ABCA1			
	ApoA1			
miR-34a	SIRT1	DNA damage and P53 pathway apoptosis and differentiation	Human osteoarthritic chondrocytes	[52]
	iNOS	Inflammation apoptosis	Rat chondrocyte culture	[53]
miR-127-5p	OPN	Cell proliferation	Human osteoarthritic chondrocytes	[59]
miR-139	MCPIP1	Inflammation	Human osteoarthritic chondrocytes	[47]
miR-140	HDAC4	Chondrocyte hypertrophy osteoblast differentiation	Murine embryos	[38]
	SOX9	Chondrogenesis	Human articular chondrocytes	[39]
	ADAMTS5	Matrix degradation	Mouse chondrocytes	[40]
	TIMP1	Matrix degradation	Mouse chondrocytes	[63]
	MMP13	Matrix degradation	Human osteoarthritic chondrocytes Mouse chondrocytes	[41, 63]
miR-146a	Bcl-2	Autophagy	C57BL/6J mouse chondrocytes	[158]
	Traf6			[159]
	IRAK1			
miR-155	Ulk1	Autophagy	Human osteoarthritic chondrocytes T/C28a2 cells	[160]
	FoxO3			
	ATG14			
	ATG5			
	ATG3			
	Gabarapl1			
	Map1lc3			
miR-449	SIRT1	Autophagy	Human osteoarthritic chondrocytes	[162]
miR-483, miR-22, miR-377, miR-103, miR-16, miR-223, miR-30b, miR-23b, miR-509, miR-29a, miR-140, miR-25, miR-337, miR-210, miR-26a, miR-373	—	—	Human osteoarthritic chondrocytes	[35]
let-7e, miRNA-454, miRNA-885-5p	—	—	Serum OA patients	[36]
miR-16, miR-20b, miR-29c, miR-30b, miR-93, miR-126, miR-146a, miR-184, miR-186, miR-195 miR-345, miR-885-5p	—	—	Plasma OA patients	[37]

## References

[1] Sarzi-Puttini P., Cimmino M. A., Scarpa R. (2005). Osteoarthritis: an overview of the disease and its treatment strategies. *Seminars in Arthritis and Rheumatism*.

[2] Wieland H. A., Michaelis M., Kirschbaum B. J., Rudolphi K. A. (2005). Osteoarthritis - an untreatable disease?. *Nature Reviews. Drug Discovery*.

[3] Felson D. T. (2006). Clinical practice. Osteoarthritis of the knee. *The New England Journal of Medicine*.

[4] Berenbaum F. (2013). Osteoarthritis as an inflammatory disease (osteoarthritis is not osteoarthrosis!). *Osteoarthritis and Cartilage*.

[5] Felson D. T., Lawrence R. C., Dieppe P. A. (2000). Osteoarthritis: new insights. Part 1: the disease and its risk factors. *Annals of Internal Medicine*.

[6] Blagojevic M., Jinks C., Jeffery A., Jordan K. P. (2010). Risk factors for onset of osteoarthritis of the knee in older adults: a systematic review and meta-analysis. *Osteoarthritis and Cartilage*.

[7] Goldring M. B., Otero M., Plumb D. A. (2011). Roles of inflammatory and anabolic cytokines in cartilage metabolism: signals and multiple effectors converge upon MMP-13 regulation in osteoarthritis. *European Cells & Materials*.

[8] van der Kraan P. M., van den Berg W. B. (2008). Osteoarthritis in the context of ageing and evolution. Loss of chondrocyte differentiation block during ageing. *Ageing Research Reviews*.

[9] Zhuo Q., Yang W., Chen J., Wang Y. (2012). Metabolic syndrome meets osteoarthritis. *Nature Reviews. Rheumatology*.

[10] Wu L., Liu H., Li L., Cheng Q., Li H., Huang H. (2014). Mitochondrial pathology in osteoarthritic chondrocytes. *Current Drug Targets*.

[11] Lopez de Figueroa P., Lotz M. K., Blanco F. J., Carames B. (2015). Autophagy activation and protection from mitochondrial dysfunction in human chondrocytes. *Arthritis Rheumatol*.

[12] Lotz M. K., Carames B. (2011). Autophagy and cartilage homeostasis mechanisms in joint health, aging and OA. *Nature Reviews. Rheumatology*.

[13] Bouderlique T., Vuppalapati K. K., Newton P. T., Li L., Barenius B., Chagin A. S. (2016). Targeted deletion of Atg5 in chondrocytes promotes age-related osteoarthritis. *Annals of the Rheumatic Diseases*.

[14] Zhang Y., Vasheghani F., Li Y. H. (2015). Cartilage-specific deletion of mTOR upregulates autophagy and protects mice from osteoarthritis. *Annals of the Rheumatic Diseases*.

[15] Loeser R. F. (2010). Age-related changes in the musculoskeletal system and the development of osteoarthritis. *Clinics in Geriatric Medicine*.

[16] Marcu K. B., Otero M., Olivotto E., Borzi R. M., Goldring M. B. (2010). NF-kappaB signaling: multiple angles to target OA. *Current Drug Targets*.

[17] Portal-Nunez S., Esbrit P., Alcaraz M. J., Largo R. (2016). Oxidative stress, autophagy, epigenetic changes and regulation by miRNAs as potential therapeutic targets in osteoarthritis. *Biochemical Pharmacology*.

[18] Neuhold L. A., Killar L., Zhao W. (2001). Postnatal expression in hyaline cartilage of constitutively active human collagenase-3 (MMP-13) induces osteoarthritis in mice. *The Journal of Clinical Investigation*.

[19] Liu-Bryan R., Terkeltaub R. (2015). Emerging regulators of the inflammatory process in osteoarthritis. *Nature Reviews. Rheumatology*.

[20] Cetrullo S., D'Adamo S., Tantini B., Borzi R. M., Flamigni F. (2015). mTOR, AMPK, and Sirt1: key players in metabolic stress management. *Critical Reviews in Eukaryotic Gene Expression*.

[21] Wu Y., Chen L., Wang Y. (2015). Overexpression of sirtuin 6 suppresses cellular senescence and NF-kappaB mediated inflammatory responses in osteoarthritis development. *Scientific Reports*.

[22] Jeffries M. A., Donica M., Baker L. W. (2014). Genome-wide DNA methylation study identifies significant epigenomic changes in osteoarthritic cartilage. *Arthritis & Rhematology*.

[23] Miyaki S., Asahara H. (2012). Macro view of microRNA function in osteoarthritis. *Nature Reviews. Rheumatology*.

[24] Goldring M. B., Berenbaum F. (2015). Emerging targets in osteoarthritis therapy. *Current Opinion in Pharmacology*.

[25] Guo H., Ingolia N. T., Weissman J. S., Bartel D. P. (2010). Mammalian microRNAs predominantly act to decrease target mRNA levels. *Nature*.

[26] Han J. J., Denli A. M., Gage F. H. (2012). The enemy within: intronic miR-26b represses its host gene, ctdsp2, to regulate neurogenesis. *Genes & Development*.

[27] Bartel D. P. (2004). MicroRNAs: genomics, biogenesis, mechanism, and function. *Cell*.

[28] Lytle J. R., Yario T. A., Steitz J. A. (2007). Target mRNAs are repressed as efficiently by microRNA-binding sites in the 5′ UTR as in the 3′ UTR. *Proceedings of the National Academy of Sciences of the United States of America*.

[29] Bartel D. P. (2009). MicroRNAs: target recognition and regulatory functions. *Cell*.

[30] Eulalio A., Behm-Ansmant I., Izaurralde E. (2007). P bodies: at the crossroads of post-transcriptional pathways. *Nature Reviews Molecular Cell Biology*.

[31] Napoli C., Lemieux C., Jorgensen R. (1990). Introduction of a chimeric chalcone synthase gene into petunia results in reversible co-suppression of homologous genes in trans. *Plant Cell*.

[32] Fire A., Xu S., Montgomery M. K., Kostas S. A., Driver S. E., Mello C. C. (1998). Potent and specific genetic interference by double-stranded RNA in *Caenorhabditis elegans*. *Nature*.

[33] Kobayashi T., Lu J., Cobb B. S. (2008). Dicer-dependent pathways regulate chondrocyte proliferation and differentiation. *Proceedings of the National Academy of Sciences of the United States of America*.

[34] Kobayashi T., Papaioannou G., Mirzamohammadi F. (2015). Early postnatal ablation of the microRNA-processing enzyme, Drosha, causes chondrocyte death and impairs the structural integrity of the articular cartilage. *Osteoarthritis and Cartilage*.

[35] Iliopoulos D., Malizos K. N., Oikonomou P., Tsezou A. (2008). Integrative microRNA and proteomic approaches identify novel osteoarthritis genes and their collaborative metabolic and inflammatory networks. *PLoS One*.

[36] Beyer C., Zampetaki A., Lin N. Y. (2014). Signature of circulating microRNAs in osteoarthritis. *Annals of the Rheumatic Diseases*.

[37] Cuadra V. M. B., Gonzalez-Huerta N. C., Romero-Cordoba S., Hidalgo-Miranda A., Miranda-Duarte A. (2014). Altered expression of circulating microRNA in plasma of patients with primary osteoarthritis and in silico analysis of their pathways. *PLoS One*.

[38] Tuddenham L., Wheeler G., Ntounia-Fousara S. (2006). The cartilage specific microRNA-140 targets histone deacetylase 4 in mouse cells. *FEBS Letters*.

[39] Miyaki S., Nakasa T., Otsuki S. (2009). MicroRNA-140 is expressed in differentiated human articular chondrocytes and modulates interleukin-1 responses. *Arthritis and Rheumatism*.

[40] Miyaki S., Sato T., Inoue A. (2010). MicroRNA-140 plays dual roles in both cartilage development and homeostasis. *Genes & Development*.

[41] Tardif G., Hum D., Pelletier J. P., Duval N., Martel-Pelletier J. (2009). Regulation of the IGFBP-5 and MMP-13 genes by the microRNAs miR-140 and miR-27a in human osteoarthritic chondrocytes. *BMC Musculoskeletal Disorders*.

[42] Gibson G., Asahara H. (2013). MicroRNAs and cartilage. *Journal of Orthopaedic Research*.

[43] Jones S. W., Watkins G., Le Good N. (2009). The identification of differentially expressed microRNA in osteoarthritic tissue that modulate the production of TNF-alpha and MMP13. *Osteoarthritis and Cartilage*.

[44] D'Adamo S., Cetrullo S., Guidotti S., Borzi R. M., Flamigni F. (2017). Hydroxytyrosol modulates the levels of microRNA-9 and its target sirtuin-1 thereby counteracting oxidative stress-induced chondrocyte death. *Osteoarthritis and Cartilage*.

[45] Song J., Kim D., Chun C. H., Jin E. J. (2013). MicroRNA-9 regulates survival of chondroblasts and cartilage integrity by targeting protogenin. *Cell Communication and Signaling*.

[46] Makki M. S., Haseeb A., Haqqi T. M. (2015). MicroRNA-9 promotion of interleukin-6 expression by inhibiting monocyte chemoattractant protein-induced protein 1 expression in interleukin-1beta-stimulated human chondrocytes. *Arthritis & Rhematology*.

[47] Makki M. S. (2015). Haqqi TM: miR-139 modulates MCPIP1/IL-6 expression and induces apoptosis in human OA chondrocytes. *Experimental & Molecular Medicine*.

[48] Akhtar N., Rasheed Z., Ramamurthy S., Anbazhagan A. N., Voss F. R., Haqqi T. M. (2010). MicroRNA-27b regulates the expression of matrix metalloproteinase 13 in human osteoarthritis chondrocytes. *Arthritis and Rheumatism*.

[49] Smith-Vikos T., Slack F. J. (2012). MicroRNAs and their roles in aging. *Journal of Cell Science*.

[50] Baker D. J., Wijshake T., Tchkonia T. (2011). Clearance of p16Ink4a-positive senescent cells delays ageing-associated disorders. *Nature*.

[51] Philipot D., Guérit D., Platano D. (2014). p16INK4a and its regulator miR-24 link senescence and chondrocyte terminal differentiation-associated matrix remodeling in osteoarthritis. *Arthritis Research & Therapy*.

[52] Yan S. J., Wang M., Zhao J. (2016). MicroRNA-34a affects chondrocyte apoptosis and proliferation by targeting the SIRT1/p53 signaling pathway during the pathogenesis of osteoarthritis. *International Journal of Molecular Medicine*.

[53] Abouheif M. M., Nakasa T., Shibuya H., Niimoto T., Kongcharoensombat W., Ochi M. (2010). Silencing microRNA-34a inhibits chondrocyte apoptosis in a rat osteoarthritis model in vitro. *Rheumatology*.

[54] Blaney Davidson E. N., van Caam A. P., van der Kraan P. M. (2017). Osteoarthritis year in review 2016: biology. *Osteoarthritis and Cartilage*.

[55] Lefort K., Brooks Y., Ostano P. (2013). A miR-34a-SIRT6 axis in the squamous cell differentiation network. *EMBO Journal*.

[56] Mostoslavsky R., Chua K. F., Lombard D. B. (2006). Genomic instability and aging-like phenotype in the absence of mammalian SIRT6. *Cell*.

[57] Nagai K., Matsushita T., Matsuzaki T. (2015). Depletion of SIRT6 causes cellular senescence, DNA damage, and telomere dysfunction in human chondrocytes. *Osteoarthritis and Cartilage*.

[58] Bai X. Y., Ma Y. X., Ding R., Fu B., Shi S. Z., Chen X. M. (2011). miR-335 and miR-34a promote renal senescence by suppressing mitochondrial antioxidative enzymes. *Journal of the American Society of Nephrology*.

[59] Tu M., Li Y., Zeng C. (2016). MicroRNA-127-5p regulates osteopontin expression and osteopontin-mediated proliferation of human chondrocytes. *Scientific Reports*.

[60] Rasheed Z., Al-Shobaili H. A., Rasheed N., Mahmood A., Khan M. I. (2016). MicroRNA-26a-5p regulates the expression of inducible nitric oxide synthase via activation of NF-kappa B pathway in human osteoarthritis chondrocytes. *Archives of Biochemistry and Biophysics*.

[61] Yin X., Wang J. Q., Yan S. Y. (2017). Reduced miR26a and miR26b expression contributes to the pathogenesis of osteoarthritis via the promotion of p65 translocation. *Molecular Medicine Reports*.

[62] Kostopoulou F., Malizos K. N., Papathanasiou I., Tsezou A. (2015). MicroRNA-33a regulates cholesterol synthesis and cholesterol efflux-related genes in osteoarthritic chondrocytes. *Arthritis Research & Therapy*.

[63] Li X. H., Zhen Z. L., Tang G. D., Zheng C., Yang G. F. (2016). MiR-29a and MiR-140 protect chondrocytes against the anti-proliferation and cell matrix signaling changes by IL-1*β*. *Molecules and Cells*.

[64] Zhu W. L., Zhao Y. L., Xu Y. Q. (2013). Dissection of protein interactomics highlights microRNA synergy. *PLoS One*.

[65] Kang L., Yang C., Song Y. (2016). MicroRNA-23a-3p promotes the development of osteoarthritis by directly targeting SMAD3 in chondrocytes. *Biochemical and Biophysical Research Communications*.

[66] Mizushima N. (2007). Autophagy: process and function. *Genes & Development*.

[67] Hohn A., Weber D., Jung T. (2016). Happily (n)ever after: aging in the context of oxidative stress, proteostasis loss and cellular senescence. *Redox Biology*.

[68] Wesselborg S., Stork B. (2015). Autophagy signal transduction by ATG proteins: from hierarchies to networks. *Cellular and Molecular Life Sciences*.

[69] Sabatini D. M. (2006). mTOR and cancer: insights into a complex relationship. *Nature Reviews. Cancer*.

[70] Sarbassov D. D., Ali S. M., Kim D. H. (2004). Rictor, a novel binding partner of mTOR, defines a rapamycin-insensitive and raptor-independent pathway that regulates the cytoskeleton. *Current Biology*.

[71] Sarbassov D. D., Guertin D. A., Ali S. M., Sabatini D. M. (2005). Phosphorylation and regulation of Akt/PKB by the rictor-mTOR complex. *Science*.

[72] Alers S., Löffler A. S., Paasch F. (2011). Atg13 and FIP200 act independently of Ulk1 and Ulk2 in autophagy induction. *Autophagy*.

[73] Liang C., Feng P., Ku B. (2006). Autophagic and tumour suppressor activity of a novel Beclin1-binding protein UVRAG. *Nature Cell Biology*.

[74] Weidberg H., Shvets E., Shpilka T., Shimron F., Shinder V., Elazar Z. (2010). LC3 and GATE-16/GABARAP subfamilies are both essential yet act differently in autophagosome biogenesis. *The EMBO Journal*.

[75] Shaid S., Brandts C. H., Serve H., Dikic I. (2013). Ubiquitination and selective autophagy. *Cell Death and Differentiation*.

[76] Levine B., Klionsky D. J. (2004). Development by self-digestion: molecular mechanisms and biological functions of autophagy. *Developmental Cell*.

[77] Egan D. F., Shackelford D. B., Mihaylova M. M. (2011). Phosphorylation of ULK1 (hATG1) by AMP-activated protein kinase connects energy sensing to Mitophagy. *Science*.

[78] Kimura N., Tokunaga C., Dalal S. (2003). A possible linkage between AMP-activated protein kinase (AMPK) and mammalian target of rapamycin (mTOR) signalling pathway. *Genes to Cells*.

[79] Lee I. H., Cao L., Mostoslavsky R. (2008). A role for the NAD-dependent deacetylase Sirt1 in the regulation of autophagy. *Proceedings of the National Academy of Sciences of the United States of America*.

[80] Mammucari C., Milan G., Romanello V. (2007). FoxO3 controls autophagy in skeletal muscle in vivo. *Cell Metabolism*.

[81] Criollo A., Maiuri M. C., Tasdemir E. (2007). Regulation of autophagy by the inositol trisphosphate receptor. *Cell Death and Differentiation*.

[82] Sarkar S., Rubinsztein D. C. (2006). Inositol and IP3 levels regulate autophagy - biology and therapeutic speculations. *Autophagy*.

[83] Galluzzi L., Pietrocola F., Levine B., Kroemer G. (2014). Metabolic control of autophagy. *Cell*.

[84] Jellinger K. A. (2010). Basic mechanisms of neurodegeneration: a critical update. *Journal of Cellular and Molecular Medicine*.

[85] Knaapen M. W., Davies M. J., De Bie M., Haven A. J., Martinet W., Kockx M. M. (2001). Apoptotic versus autophagic cell death in heart failure. *Cardiovascular Research*.

[86] Kirshenbaum L. A. (2012). Regulation of autophagy in the heart in health and disease. *Journal of Cardiovascular Pharmacology*.

[87] Cetrullo S., Tantini B., Flamigni F. (2012). Antiapoptotic and antiautophagic effects of eicosapentaenoic acid in cardiac myoblasts exposed to palmitic acid. *Nutrients*.

[88] White E. (2012). Deconvoluting the context-dependent role for autophagy in cancer. *Nature Reviews. Cancer*.

[89] Roach H. I., Aigner T., Kouri J. B. (2004). Chondroptosis: a variant of apoptotic cell death in chondrocytes?. *Apoptosis*.

[90] Almonte-Becerril M., Navarro-Garcia F., Gonzalez-Robles A., Vega-Lopez M. A., Lavalle C., Kouri J. B. (2010). Cell death of chondrocytes is a combination between apoptosis and autophagy during the pathogenesis of osteoarthritis within an experimental model. *Apoptosis*.

[91] Rubinsztein D. C., Codogno P., Levine B. (2012). Autophagy modulation as a potential therapeutic target for diverse diseases. *Nature Reviews. Drug Discovery*.

[92] Carames B., Kiosses W. B., Akasaki Y. (2013). Glucosamine activates autophagy in vitro and in vivo. *Arthritis and Rheumatism*.

[93] Matsuzaki T., Matsushita T., Tabata Y. (2014). Intra-articular administration of gelatin hydrogels incorporating rapamycin-micelles reduces the development of experimental osteoarthritis in a murine model. *Biomaterials*.

[94] Borzì R. M., Guidotti S., Minguzzi M. (2014). Polyamine delivery as a tool to modulate stem cell differentiation in skeletal tissue engineering. *Amino Acids*.

[95] Sasaki H., Takayama K., Matsushita T. (2012). Autophagy modulates osteoarthritis-related gene expression in human chondrocytes. *Arthritis and Rheumatism*.

[96] Takayama K., Kawakami Y., Kobayashi M. (2014). Local intra-articular injection of rapamycin delays articular cartilage degeneration in a murine model of osteoarthritis. *Arthritis Research & Therapy*.

[97] Carames B., Taniguchi N., Otsuki S., Blanco F. J., Lotz M. (2010). Autophagy is a protective mechanism in normal cartilage, and its aging-related loss is linked with cell death and osteoarthritis. *Arthritis and Rheumatism*.

[98] Alvarez-Garcia O., Olmer M., Akagi R. (2016). Suppression of REDD1 in osteoarthritis cartilage, a novel mechanism for dysregulated mTOR signaling and defective autophagy. *Osteoarthritis and Cartilage*.

[99] Zhang H., Wang H., Zeng C. (2017). mTORC1 activation downregulates FGFR3 and PTH/PTHrP receptor in articular chondrocytes to initiate osteoarthritis. *Osteoarthritis and Cartilage*.

[100] Vasheghani F., Zhang Y., Li Y. H. (2015). PPARgamma deficiency results in severe, accelerated osteoarthritis associated with aberrant mTOR signalling in the articular cartilage. *Annals of the Rheumatic Diseases*.

[101] Lee J. H., Budanov A. V., Karin M. (2013). Sestrins orchestrate cellular metabolism to attenuate aging. *Cell Metabolism*.

[102] Shen T., Alvarez-Garcia O., Li Y., Olmer M., Lotz M. K. (2017). Suppression of Sestrins in aging and osteoarthritic cartilage: dysfunction of an important stress defense mechanism. *Osteoarthritis and Cartilage*.

[103] Ribeiro M., Lopez de Figueroa P., Blanco F. J., Mendes A. F., Carames B. (2016). Insulin decreases autophagy and leads to cartilage degradation. *Osteoarthritis and Cartilage*.

[104] Ribeiro M., Lopez de Figueroa P., Nogueira-Recalde U. (2016). Diabetes-accelerated experimental osteoarthritis is prevented by autophagy activation. *Osteoarthritis and Cartilage*.

[105] Dumond H., Presle N., Terlain B. (2003). Evidence for a key role of leptin in osteoarthritis. *Arthritis and Rheumatism*.

[106] Huang Z. M., Du S. H., Huang L. G., Li J. H., Xiao L., Tong P. (2016). Leptin promotes apoptosis and inhibits autophagy of chondrocytes through upregulating lysyl oxidase-like 3 during osteoarthritis pathogenesis. *Osteoarthritis and Cartilage*.

[107] Terkeltaub R., Yang B., Lotz M., Liu-Bryan R. (2011). Chondrocyte AMP-activated protein kinase activity suppresses matrix degradation responses to proinflammatory cytokines interleukin-1 beta and tumor necrosis factor alpha. *Arthritis and Rheumatism*.

[108] Liu-Bryan R. (2015). Inflammation and intracellular metabolism: new targets in OA. *Osteoarthritis and Cartilage*.

[109] Shen C., Cai G. Q., Peng J. P., Chen X. D. (2015). Autophagy protects chondrocytes from glucocorticoids-induced apoptosis via ROS/Akt/FOXO3 signaling. *Osteoarthritis and Cartilage*.

[110] Shen C., Gu W., Cai G. Q., Peng J. P., Chen X. D. (2015). Autophagy protects meniscal cells from glucocorticoids-induced apoptosis via inositol trisphosphate receptor signaling. *Apoptosis*.

[111] Castrogiovanni P., Trovato F. M., Loreto C., Nsir H., Szychlinska M. A., Musumeci G. (2016). Nutraceutical supplements in the management and prevention of osteoarthritis. *International Journal of Molecular Sciences*.

[112] Facchini A., Stanic I., Cetrullo S., Borzi R. M., Filardo G., Flamigni F. (2011). Sulforaphane protects human chondrocytes against cell death induced by various stimuli. *Journal of Cellular Physiology*.

[113] Facchini A., Cetrullo S., D'Adamo S. (2014). Hydroxytyrosol prevents increase of osteoarthritis markers in human chondrocytes treated with hydrogen peroxide or growth-related oncogene alpha. *PLoS One*.

[114] Cetrullo S., D'Adamo S., Guidotti S., Borzi R. M., Flamigni F. (2016). Hydroxytyrosol prevents chondrocyte death under oxidative stress by inducing autophagy through sirtuin 1-dependent and -independent mechanisms. *Biochimica et Biophysica Acta*.

[115] Flamigni F., Stanic I., Facchini A. (2007). Polyamine biosynthesis as a target to inhibit apoptosis of non-tumoral cells. *Amino Acids*.

[116] Stanic I., Cetrullo S., Facchini A. (2008). Effect of the polyamine analogue N-1,N-11-diethylnorspermine on cell survival and susceptibility to apoptosis of human chondrocytes. *Journal of Cellular Physiology*.

[117] Facchini A., Borzi R. M., Olivotto E. (2012). Role of polyamines in hypertrophy and terminal differentiation of osteoarthritic chondrocytes. *Amino Acids*.

[118] Eisenberg T., Knauer H., Schauer A. (2009). Induction of autophagy by spermidine promotes longevity. *Nature Cell Biology*.

[119] Ghisalberti C. A., Borzi R. M., Cetrullo S., Flamigni F., Cairo G. (2016). Soft TCPTP agonism-novel target to rescue airway epithelial integrity by exogenous spermidine. *Frontiers in Pharmacology*.

[120] Pietrocola F., Lachkar S., Enot D. P. (2015). Spermidine induces autophagy by inhibiting the acetyltransferase EP300. *Cell Death and Differentiation*.

[121] Decressac M., Mattsson B., Weikop P., Lundblad M., Jakobsson J., Bjorklund A. (2013). TFEB-mediated autophagy rescues midbrain dopamine neurons from alpha-synuclein toxicity. *Proceedings of the National Academy of Sciences of the United States of America*.

[122] Xu J. Z., Wang Y. F., Tan X. R., Jing H. J. (2012). MicroRNAs in autophagy and their emerging roles in crosstalk with apoptosis. *Autophagy*.

[123] Frankel L. B., Lubas M., Lund A. H. (2017). Emerging connections between RNA and autophagy. *Autophagy*.

[124] Zhu H., Wu H., Liu X. P. (2009). Regulation of autophagy by a Beclin 1-targeted microRNA, miR-30a, in cancer cells. *Autophagy*.

[125] Chen Y., Liersch R., Detmar M. (2012). The miR-290-295 cluster suppresses autophagic cell death of melanoma cells. *Scientific Reports*.

[126] Wu H., Wang F. L., Hu S. L. (2012). MiR-20a and miR-106b negatively regulate autophagy induced by leucine deprivation via suppression of *ULK1* expression in C2C12 myoblasts. *Cellular Signalling*.

[127] Wang Z., Wang N., Liu P. (2014). MicroRNA-25 regulates chemoresistance-associated autophagy in breast cancer cells, a process modulated by the natural autophagy inducer isoliquiritigenin. *Oncotarget*.

[128] Huang Y., Chuang A. Y., Ratovitski E. A. (2011). Phospho-ΔNp63α/miR-885-3p axis in tumor cell life and cell death upon cisplatin exposure. *Cell Cycle*.

[129] Tang B., Li N., Gu J. (2012). Compromised autophagy by MIR30B benefits the intracellular survival of Helicobacter pylori. *Autophagy*.

[130] Korkmaz G., le Sage C., Tekirdag K. A., Agami R., Gozuacik D. (2012). miR-376b controls starvation and mTOR inhibition-related autophagy by targeting ATG4C and BECN1. *Autophagy*.

[131] Chatterjee A., Chattopadhyay D., Chakrabarti G. (2014). miR-17-5p downregulation contributes to paclitaxel resistance of lung cancer cells through altering Beclin1 expression. *PLoS One*.

[132] Menghini R., Casagrande V., Marino A. (2014). MiR-216a: a link between endothelial dysfunction and autophagy. *Cell Death & Disease*.

[133] He J., Yu J. J., Xu Q. (2015). Downregulation of ATG14 by EGR1-MIR152 sensitizes ovarian cancer cells to cisplatin-induced apoptosis by inhibiting cyto-protective autophagy. *Autophagy*.

[134] Frankel L. B., Wen J. Y., Lees M. (2011). microRNA-101 is a potent inhibitor of autophagy. *EMBO Journal*.

[135] Pan B. Z., Chen Y. T., Song H. Z., Xu Y. C., Wang R., Chen L. B. (2015). Mir-24-3p downregulation contributes to VP16-DDP resistance in small-cell lung cancer by targeting ATG4A. *Oncotarget*.

[136] Liu X. J., Hong Q., Wang Z., Yu Y. Y., Zou X., Xu L. H. (2015). MicroRNA-34a suppresses autophagy in tubular epithelial cells in acute kidney injury. *American Journal of Nephrology*.

[137] Hall D. P., Cost N. G., Hegde S. (2014). TRPM3 and miR-204 establish a regulatory circuit that controls oncogenic autophagy in clear cell renal cell carcinoma. *Cancer Cell*.

[138] Mikhaylova O., Stratton Y., Hall D. (2012). VHL-regulated MiR-204 suppresses tumor growth through inhibition of LC3B-mediated autophagy in renal clear cell carcinoma. *Cancer Cell*.

[139] Du F. J., Feng Y. X., Fang J. Z., Yang M. W. (2015). MicroRNA-143 enhances chemosensitivity of quercetin through autophagy inhibition via target GABARAPL1 in gastric cancer cells. *Biomedicine & Pharmacotherapy*.

[140] Yu Y., Yang L., Zhao M. (2012). Targeting microRNA-30a-mediated autophagy enhances imatinib activity against human chronic myeloid leukemia cells. *Leukemia*.

[141] Tekirdag K. A., Korkmaz G., Ozturk D. G., Agami R., Gozuacik D. (2013). MIR181A regulates starvation- and rapamycin-induced autophagy through targeting of ATG5. *Autophagy*.

[142] Guo X., Xue H., Guo X. F. (2015). MiR224-3p inhibits hypoxia-induced autophagy by targeting autophagy-related genes in human glioblastoma cells. *Oncotarget*.

[143] Meenhuis A., van Veelen P. A., de Looper H. (2011). MiR-17/20/93/106 promote hematopoietic cell expansion by targeting sequestosome 1-regulated pathways in mice. *Blood*.

[144] Okato A., Goto Y., Kurozumi A. (2016). Direct regulation of LAMP1 by tumor-suppressive microRNA-320a in prostate cancer. *International Journal of Oncology*.

[145] Frankel L. B., Di Malta C., Wen J. Y., Eskelinen E. L., Ballabio A., Lund A. H. (2014). A non-conserved miRNA regulates lysosomal function and impacts on a human lysosomal storage disorder. *Nature Communications*.

[146] Wan G., Xie W., Liu Z. (2014). Hypoxia-induced *MIR155* is a potent autophagy inducer by targeting multiple players in the MTOR pathway. *Autophagy*.

[147] Ge Y. Y., Shi Q., Zheng Z. Y. (2014). MicroRNA-100 promotes the autophagy of hepatocellular carcinoma cells by inhibiting the expression of mTOR and IGF-1R. *Oncotarget*.

[148] Dubinsky A. N., Dastidar S. G., Hsu C. L. (2014). Let-7 coordinately suppresses components of the amino acid sensing pathway to repress mTORC1 and induce autophagy. *Cell Metabolism*.

[149] Mavrakis K. J., Wolfe A. L., Oricchio E. (2010). Genome-wide RNA-mediated interference screen identifies miR-19 targets in notch-induced T-cell acute lymphoblastic leukaemia. *Nature Cell Biology*.

[150] Li J., Li J. F., Wei T. T., Li J. H. (2016). Down-regulation of microRNA-137 improves high glucose-induced oxidative stress injury in human umbilical vein endothelial cells by up-regulation of AMPK alpha 1. *Cellular Physiology and Biochemistry*.

[151] Yamakuchi M., Ferlito M., Lowenstein C. J. (2008). miR-34a repression of SIRT1 regulates apoptosis. *Proceedings of the National Academy of Sciences of the United States of America*.

[152] Ramalinga M., Roy A., Srivastava A. (2015). MicroRNA-212 negatively regulates starvation induced autophagy in prostate cancer cells by inhibiting SIRT1 and is a modulator of angiogenesis and cellular senescence. *Oncotarget*.

[153] Yang Y., Liu Y., Xue J. (2017). MicroRNA-141 targets Sirt1 and inhibits autophagy to reduce HBV replication. *Cellular Physiology and Biochemistry*.

[154] Mehta A., Zhao J. L., Sinha N. (2015). The microRNA-132 and microRNA-212 cluster regulates hematopoietic stem cell maintenance and survival with age by buffering FOXO3 expression. *Immunity*.

[155] Chen Z., Lin T., Lu Y. (2016). AntimiR-30b inhibits TNF-alpha mediated apoptosis and attenuated cartilage degradation through enhancing autophagy. *Cellular Physiology and Biochemistry*.

[156] Lewis B. P., Shih I. H., Jones-Rhoades M. W., Bartel D. P., Burge C. B. (2003). Prediction of mammalian microRNA targets. *Cell*.

[157] Xu K., Xu P., Yao J. F., Zhang Y. G., Hou W. K., Lu S. M. (2013). Reduced apoptosis correlates with enhanced autophagy in synovial tissues of rheumatoid arthritis. *Inflammation Research*.

[158] Zhang F., Wang J., Chu J. (2015). MicroRNA-146a induced by hypoxia promotes chondrocyte autophagy through BcI-2. *Cellular Physiology and Biochemistry*.

[159] Chen G., Gao X., Wang J. (2017). Hypoxia-induced microRNA-146a represses Bcl-2 through Traf6/IRAK1 but not Smad4 to promote chondrocyte autophagy. *Biological Chemistry*.

[160] D'Adamo S., Alvarez-Garcia O., Muramatsu Y., Flamigni F., Lotz M. K. (2016). MicroRNA-155 suppresses autophagy in chondrocytes by modulating expression of autophagy proteins. *Osteoarthritis and Cartilage*.

[161] Weilner S., Grillari-Voglauer R., Redl H., Grillari J., Nau T. (2015). The role of microRNAs in cellular senescence and age-related conditions of cartilage and bone. *Acta Orthopaedica*.

[162] Park K. W., Lee K. M., Yoon D. S. (2016). Inhibition of microRNA-449a prevents IL-1beta-induced cartilage destruction via SIRT1. *Osteoarthritis and Cartilage*.

[163] Song J., Ahn C., Chun C. H., Jin E. J. (2014). A long non-coding RNA, GAS5, plays a critical role in the regulation of miR-21 during osteoarthritis. *Journal of Orthopaedic Research*.

[164] Gibbings D., Mostowy S., Jay F., Schwab Y., Cossart P., Voinnet O. (2012). Selective autophagy degrades *DICER* and *AGO2* and regulates miRNA activity. *Nature Cell Biology*.

[165] Li Z., Rana T. M. (2014). Therapeutic targeting of microRNAs: current status and future challenges. *Nature Reviews. Drug Discovery*.

